# Severe polyarterial involvement in a 16-year-old with homozygous familial hypercholesterolaemia: a case report

**DOI:** 10.1093/ehjcr/ytag227

**Published:** 2026-03-26

**Authors:** Malak Benabdellah, Mohammed Bachir Mesfioui, Hajar El Ouartassi, Badre El Boussaadani, Zainab Raissuni

**Affiliations:** Cardiology Department, Mohammed VI University Hospital, Tangier 90000, Morocco; Cardiology Department, Mohammed VI University Hospital, Tangier 90000, Morocco; Cardiology Department, Mohammed VI University Hospital, Tangier 90000, Morocco; Cardiology Department, Mohammed VI University Hospital, Tangier 90000, Morocco; Faculty of Medicine and Pharmacy of Tangier, Abdelmalek Essadi University, Tangier 90000, Morocco; Cardiology Department, Mohammed VI University Hospital, Tangier 90000, Morocco; Faculty of Medicine and Pharmacy of Tangier, Abdelmalek Essadi University, Tangier 90000, Morocco

**Keywords:** Familial hypercholesterolaemia, Case report, Low-density-lipoprotein, Atherosclerosis, Lipid-lowering therapy

## Abstract

**Background:**

Homozygous familial hypercholesterolaemia (HoFH) is a rare genetic disorder characterized by an elevated plasma concentration of low-density lipoprotein cholesterol (LDL-C) starting at birth and a significantly increased risk of premature atherosclerotic cardiovascular disease.

**Case summary:**

We report the case of a 16-year-old female patient, with no known consanguinity, presented to our cardiology department for anginal chest pain on exertion associated with headaches. She presented with characteristic morphological features of FH. Her lipid profile revealed extremely high LDL-C levels (706 mg/dL) and such extensive arterial and cutaneous involvement.

**Discussion:**

This case underscores the importance of recognizing xanthomas and their association with an increased risk of coronary atherosclerosis.

Learning points

- Hypercholesterolaemia is a fatal, rare, life-threatening, and genetic disorder, characterized by elevated levels of low-density lipoprotein cholesterol from birth, xanthoma, and accelerated atherosclerosis.
- If left untreated, it leads to premature cardiovascular morbidity and mortality; hence, early identification and prompt initiation of lipid-lowering therapies are essential.

## Introduction

Familial hypercholesterolaemia (FH) is an inherited cause of very high serum cholesterol, present from birth, due to mutations affecting low-density lipoprotein receptor (LDLR) activity.^1^ Homozygous familial hypercholesterolaemia (HoFH) is a rare and life-threatening condition, initially characterized by plasma cholesterol levels >13 mmol/L (>500 mg/dL), extensive xanthomas, and marked premature, progressive atherosclerotic cardiovascular disease (ACVD). If left untreated, most patients with markedly elevated LDL-C levels develop atherosclerosis before the age of 20 years, and rarely survive beyond the age of 30.^2^ This case highlights the challenges of lowering LDL-C levels and preventing related complications, emphasizing that not only lowering, but early lowering is better.

## Summary figure

**Figure:**
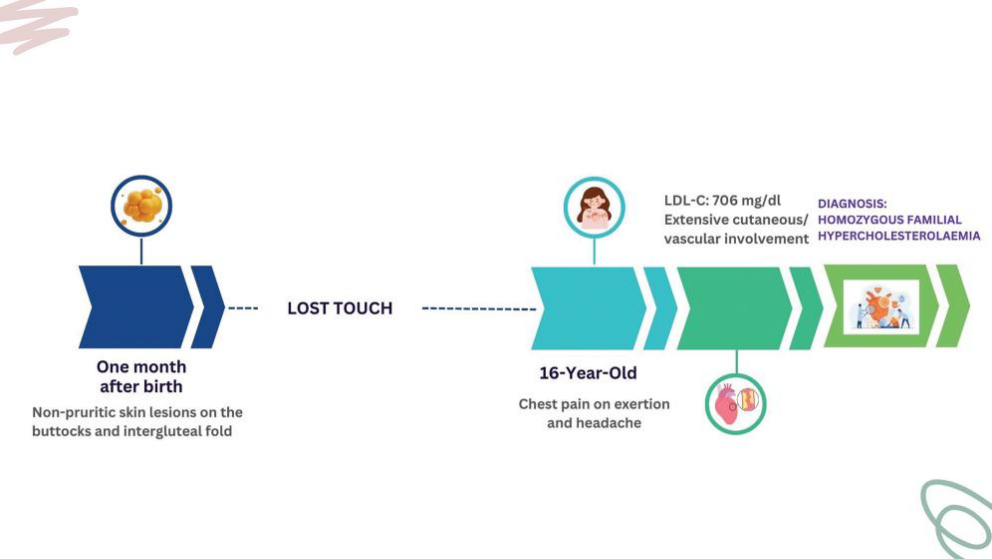


## Case presentation

A 16-year-old female patient with no known consanguinity presented to our cardiology department for anginal chest pain on exertion associated with headaches. Her personal medical history was unremarkable, with no diabetes, hypertension, or known hepatic disorders. Physical examination revealed a BMI of 19 kg/m^2^ and no congestive heart failure, and cardiac and pulmonary auscultation was unremarkable.

The electrocardiogram showed a regular rhythm, no ventricular hypertrophy, and no conduction disorder. The troponin level was normal. Transthoracic echocardiography did not show any cardiomyopathy or significant valvular disease.

**Figure 1:**
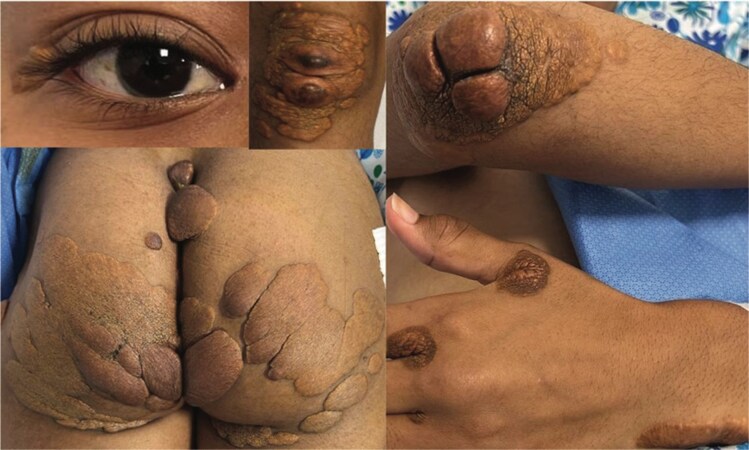
Our patient’s skin lesions.

**Figure 2:**
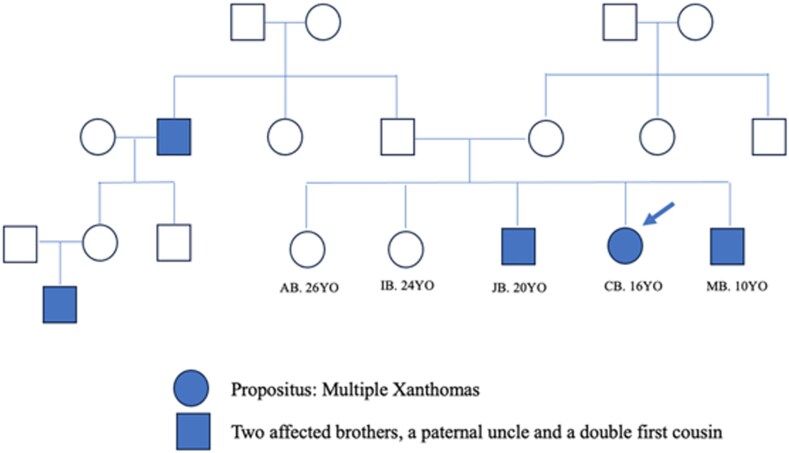
Family tree.

**Figure 3:**
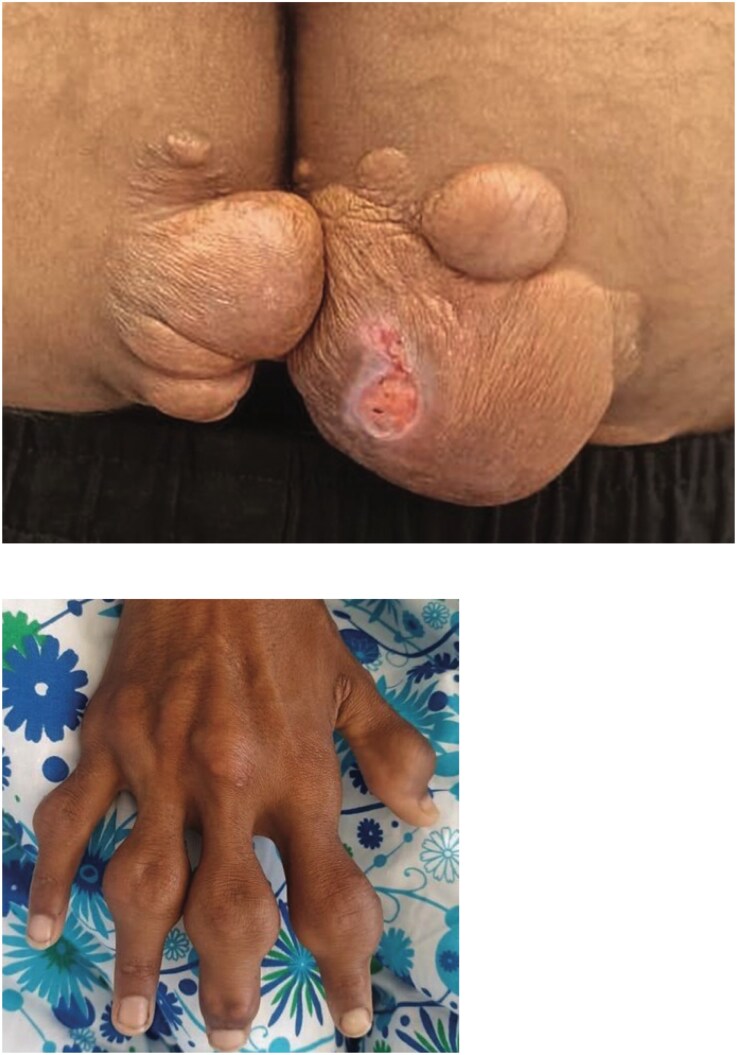
Her brother, with LDL-C at 600 mg/dL, had bilateral 70% carotid artery stenosis and subclavian artery involvement. He is still undergoing evaluation.

**Figure 4:**
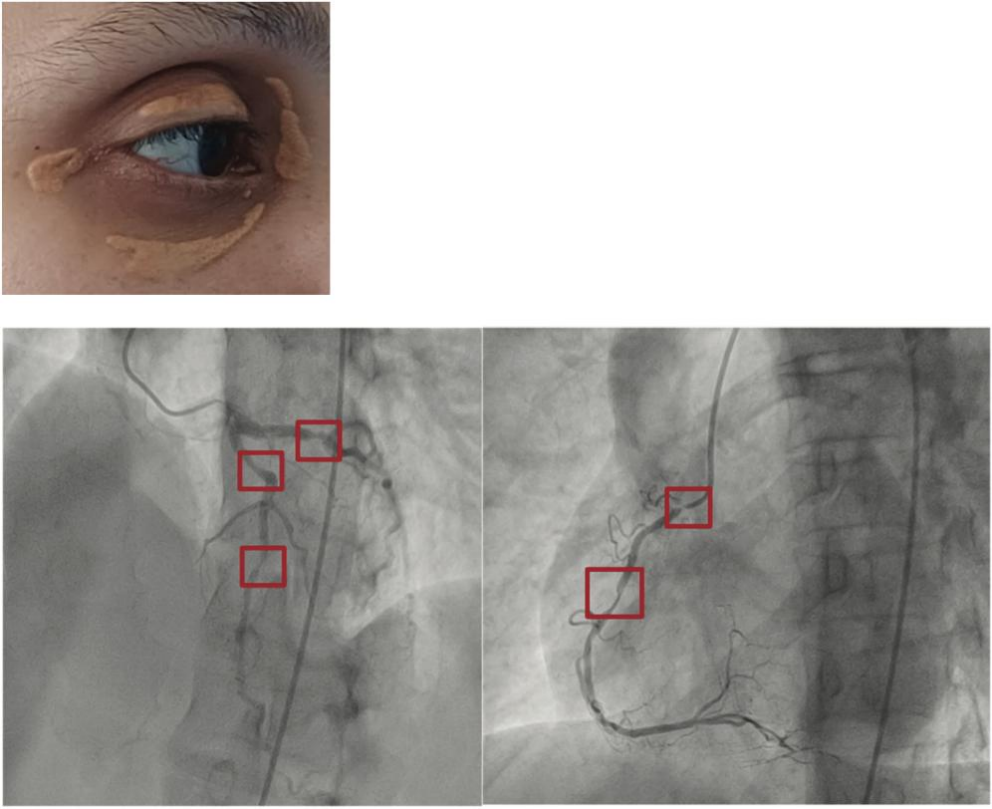
Her cousin, whose LDL-C was 486 mg/dL, and angiography revealed: a subocclusive proximal RCA lesion. A severe mid-RCA stenosis. A significant proximal circumflex stenosis. A 70% mid-LAD stenosis.

Inspections revealed multiple cutaneous firm, painless, brown-yellow papules and plaques over the interdigital areas, elbows, knees, ankles, buttocks, and eyelids. Some lesions had a rough surface. Corneal arcus (gerontoxon) was noted (*Figure 1*). The mother reported that these conditions were first noticed one month after birth, with nonpruritic skin lesions on the buttocks. Over the years, these lesions gradually spread to the knees, ankles, elbows, fingers, shoulders, neck, and eyelids without affecting her general health. She described similar skin lesions affecting two brothers and a paternal cousin (*Figures 2–4*). The patient’s lipid panel revealed a hypercholesterolaemia with LDL-C: 18.3 mmol/L and HDL-C: 0.72 mmol/L. No hypothyroidism, nephrotic syndrome, or renal impairment.

**Figure 5:**
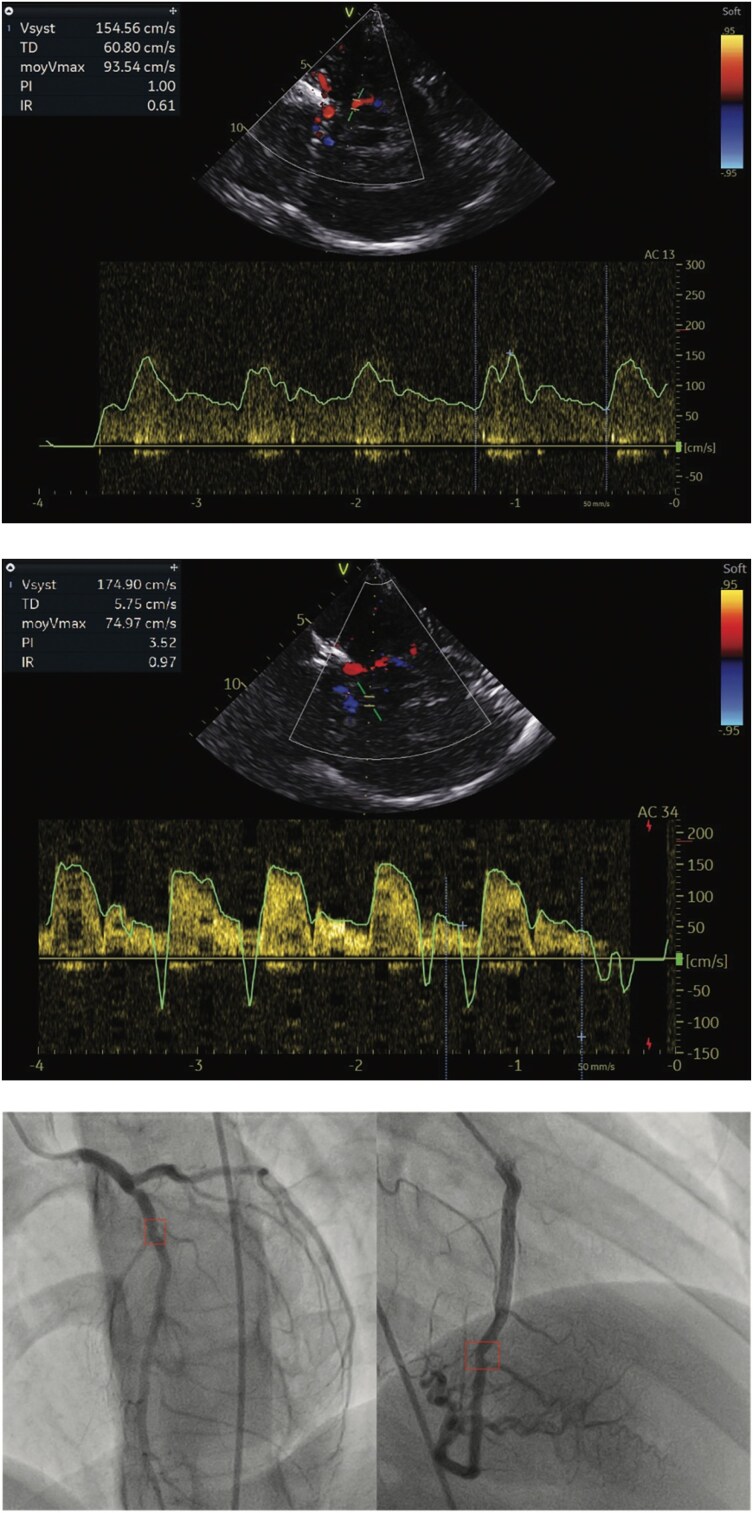
Transcranial Doppler revealed increased flow velocities in the middle cerebral artery. Image of coronary lesions.

We, therefore, conducted additional investigations, including a CT angiography of the cerebral arteries/supra-aortic trunks and a transcranial doppler ultrasound, which revealed a diffuse bilateral atherosclerotic burden, more pronounced in the left internal carotid artery, leading to a reduced lumen and complete stenosis at its intracavernous portion, with revascularization downstream. Circumferential, nonstenotic, partially calcified atherosclerosis of the aortic arch and ascending aorta. The right subclavian artery shows an atheromatous plaque at its proximal segment, measuring 3 mm thick and extending over 13 cm, reducing the lumen by 45%. Widespread atherosclerotic thickening along the common and internal cervical carotid arteries. Coronary angiography findings showed: A proximal lesion of the left anterior descending artery with less than 50% stenosis. A 30% stenotic lesion in the mid-right coronary artery (*Figure 5*) genetic testing: Sequencing of exon 6 of the LDLR gene did not identify any mutations. A comprehensive NGS panel for dyslipidaemia has been requested for further analysis. The diagnosis of HoFH was based on clinical presentation and biological results, scoring 22 points on the Dutch Lipid Clinic Network.

The patient was started on Rosuvastatin 40 mg and Ezetimibe 10 mg OD. Her coronary stenosis and vascular disease were medically managed: Oral aspirin was administered 75 mg once daily. She was advised to monitor her lipid profile and family screening regularly. We envisaged a clinical evaluation of her cardiovascular disease every 6 months and immediate investigation in the event of cardiovascular symptoms, like angina or the appearance of an equivalent condition, such as shortness of breath, easy fatigue, tachyarrhythmia, or postural syncope, which may indicate aortic stenosis. Additional tests to detect asymptomatic cardiac ischaemia should be considered every 5 years, or for symptomatic evaluation, if necessary, by coronary computed tomography or cardiac magnetic resonance imaging; if not available, an exercise test such as stress echocardiography. During follow-up, LDL levels began to decrease but remained at the upper limit of the normal range. We are considering initiating PCSK9 inhibitor therapy abroad due to its unavailability in the patient’s country of origin.

## Discussion

Over 90% of genetically confirmed FH cases are due to biallelic/likely pathogenic variants on different chromosomes at the LDL receptor *(LDLR*) genes or ≥2 such variants at different loci. It is also caused by mutations in genes encoding apolipoprotein B (*APOB*), proprotein convertase subtilisin-kexin type 9 (*PCSK9*), or LDL receptor adaptor protein 1 (*LDLRAP1*).^3,4^ Based on 2020 population data and estimates of homozygous familial hypercholesterolaemia prevalence ranging from 1:250 000 to 1:360 000, underdiagnosis and undertreatment of HoFH are major issues impacting HoFH care; recent estimates indicate that ∼30 000 people worldwide have HoFH, but <5% are identified.^4^ The diagnosis can be made based on genetic or clinical criteria. While genetic testing may provide a definitive diagnosis of HoFH, it is recognized that in some patients, genetic confirmation remains elusive, despite exhaustive investigation; indeed, the existence of additional FH genes cannot be excluded. HoFH has been most commonly diagnosed based on clinical criteria: an untreated LDL-C plasma concentration >10 mmol/L (>400 mg/dL) and the presence of cutaneous or tendon xanthomas before the age of 10 years, or the presence of untreated elevated LDL-C levels consistent with HoFH in both parents and/or genetic testing.^2,4,5^

The early identification of these patients and prompt referral to a specialized clinic is crucial. In primary prevention, for individuals with FH at very-high risk, an LDL-C reduction of ≥50% from baseline and an LDL-C goal of <1.4 mmol/L (<55 mg/dL) should be considered.^6^ All registries document the difficulty of achieving treatment goals for LDL-cholesterol defined by existing evidence-based guidelines; for that, the aim is to start treatment, beginning with rapid uptitration of statins and ezetimibe, followed by introduction of specialized treatment, when available, with lipoprotein apheresis. This treatment (every 1–2 weeks) can decrease plasma LDL-C levels by 55%–70%. The procedure frequency may be adjusted for each patient as lipid levels, symptoms, and other disease-related parameters change. Maximally tolerated pharmacological therapy must be maintained. Treatment with a PCSK9 inhibitor is recommended in very-high-risk FH patients if the treatment goal is not achieved on maximal tolerated statin plus ezetimibe.^4,6,7^

Lipoprotein apheresis remains an essential form of treatment for some HoFH patients, as recently affirmed by the European Atherosclerosis Society and by the American Heart Association.^4^ This position has also been supported by the European Society of Cardiology’s latest guidelines^6^ on lipoprotein cholesterol lowering by including new agents for LDL C reducing agents such as bempedoic acid and evinacumab, a monoclonal antibody that inhibits angiopoietin-like-3 (ANGPTL3), specifically for patients with HoFH. Other sources have also demonstrated the efficacy of lomitapide, a small-molecule inhibitor of the microsomal triglyceride transfer protein (MTP). By directly binding to MTP in the endoplasmic reticulum of hepatocytes and enterocytes, lomitapide inhibits the assembly of VLDL and chylomicrons, thereby offering an additional therapeutic strategy for HoFH.^8^ However, both lomitapide and evinacumab are very expensive and therefore may not be viable options for many health care systems, notably in our case. The effect of combining multiple lipid-lowering therapies such as statins, ezetimibe, PCSK9 inhibitors, and lomitapide is limited in severe HoFH patients with null/null pathogenic variants in *LDLR*.^5,9^

Moreover, intensive LDL cholesterol lowering is advocated in current clinical guidelines to slow down plaque progression and lower the risk of premature cardiovascular events.^10^ This has been proven. In patients with homozygous familial hypercholesterolemia receiving maximum doses of lipid-lowering therapy, the reduction from baseline in the LDL cholesterol level in the evinacumab group, compared with the small increase in the placebo group, was observed at 24 weeks.^11^ Additionally, two young HoFH patients in whom profound plaque reduction was observed with CCTA imaging after highly intensive lipid-lowering therapy with statins, ezetimibe, LDL apheresis, and evinacumab.^10^

However, an unacceptably high number of children and young adults living with xantomas and HoFH remain undiagnosed, misdiagnosed, and receive a late diagnosis, often after a major cardiovascular event. New hope exists for those with HoFH; cascade screening of first-degree and second-degree relatives is important because early treatment extends life expectancy substantially.^12^

Our case highlights the importance of raising awareness about detecting skin lesions associated with HoFH from birth. Several treatments are currently available, and advances in genetics enable the selection of the most appropriate therapy for each patient. Cardiac imaging also plays a key role in closely monitoring these patients. Above all, treatment should be initiated as early as possible to prevent the development of subsequent arterial lesions.

## Conclusion

Familial hypercholesterolaemia is a rare disorder with limited data available. It remains widely underdiagnosed and often undertreated. The choice of therapy, the optimal approach, and timing are crucial to limiting arterial involvement.

## Data Availability

The data underlying this article are available in the article.

## Lead author biography

Benabdellah Malak is a Cardiology resident at the Cardiology Department of the University Hospital in Tangier, Morocco. In addition to her daily clinical activities, her main areas of interest focus on hypertension, acute heart failure with cardiogenic shock, with a particular interest in cardiac intensive care.

## Notes

**Consent:** The authors confirm that written consent for submission and publication of this case report including imaging and associated text has been obtained from the patient in line with COPE guidance.
